# In-hospital outcomes among patients admitted for myocardial infarction with coexisting respiratory syncytial virus versus influenza infections

**DOI:** 10.1007/s11739-026-04335-9

**Published:** 2026-03-31

**Authors:** Conner E. Johnson, Whitney B. Sussman, Erin R. Weeda

**Affiliations:** University of South Carolina School of Medicine Greenville, 607 Grove Rd, Greenville, SC USA

**Keywords:** Myocardial infarction, Respiratory syncytial virus infections, Influenza, Human, Cardiovascular diseases, Viral infections

## Abstract

Among patients with myocardial infarction, influenza infection increases the risk of severe complications and mortality, likely driven by combined inflammatory and cardiovascular stress. Little is known about coexisting respiratory syncytial virus (RSV) infection among those presenting with myocardial infarction. This study aimed to investigate whether in-hospital outcomes differ among patients admitted for myocardial infarction with coexisting RSV versus influenza infections. Using the 2016–2022 United States National Inpatient Sample, we identified all individuals admitted for myocardial infarction. Only those with coexisting RSV or influenza infections were included. In-hospital outcomes were compared among those with RSV versus influenza. We identified 3966 patients with myocardial infarction in the database meeting our inclusion criteria, of which 565 had coexisting RSV and 3401 had coexisting influenza infections. In-hospital mortality occurred in 5.7% of individuals with RSV and 5.8% of individuals with influenza infections. The average length of hospital stay in all patients was approximately 7 days for both those with RSV and those with influenza. After adjusting for covariates, there was no difference in mortality (odds ratio [OR] = 0.925;95% confidence interval [CI] = 0.620–1.382), in-hospital complications (OR = 0.973;95%CI = 0.738–1.282), or LOS (mean difference = 0.17; 95%CI = − 0.30 to 0.64). In conclusion, this study found no significant difference in in-hospital mortality or length of stay between patients with myocardial infarction coexisting with RSV versus influenza infections. Given the established role of influenza in exacerbating myocardial infarction severity, greater recognition and investigation of RSV co-infections in this high-risk population are likely needed.

## Introduction

Influenza has long been associated with increased rates of acute cardiovascular events [1]. Respiratory syncytial virus (RSV) infections are increasingly being recognized as a health risk in the elderly as well as in those with comorbidities [2]. Given the association between acute respiratory infections and cardiovascular events, RSV infection would be expected to impact cardiovascular-related outcomes for some patients [1]. However, little is known about in-hospital outcomes among patients with both acute MI events and RSV infections. This study aimed to investigate whether in-hospital outcomes differ among patients admitted for MI with coexisting RSV versus influenza infections.

## Methods

We identified adult patients treated at US hospitals for acute MI between January 1, 2016 and December 31, 2022 using the Healthcare Cost and Utilization Project (HCUP) National Inpatient Sample (NIS) claims database [3]. All consecutively admitted patients were included, including those transferred from other facilities. The NIS is the largest all-payer inpatient healthcare database accessible to the public in the United States. It contains data from about 7 million hospitalizations each year.

Encounters were included if they had an ICD-10CM code indicating acute MI in the primary diagnostic position (Supplemental Table 1) [4]. The cohort was restricted to individuals with a coexisting RSV or influenza infection. RSV and influenza infections were identified using the ICD-10CM codes shown in Supplemental Table 1 [3–8]. Included patients were divided into two cohorts based on the coexistence of RSV or influenza. Patients with both RSV and influenza infections simultaneously were excluded from the analysis. Demographics, comorbidities, and hospital characteristics were compared between cohorts. Outcomes of interest included in-hospital mortality, in-hospital complications (which were a composite of cardiogenic shock, sepsis, mechanical ventilation, and cardiac arrest), and hospital length of stay (LOS). Several sensitivity and subgroup analyses were performed. First, we divided included patients into those that presented prior to versus after 2020. Second, we excluded patients with concomitant COVID-19 infections.

**Table 1:** Characteristics of myocardial infarction patients with concomitant respiratory syncytial virus and influenza infection

	RSV *N* = 565 *n* (%)	Influenza *N* = 3401 *n* (%)	*P*
Age, years, median (IQR)	74 (65–82)	72 (63–81)	0.002
Age ≥ 75 years	276 (48.8)	1455 (42.8)	0.007
Male	271 (48.0)	1877 (55.2)	0.001
White	399 (70.6)	2272 (66.8)	0.073
≥ 3 Elixhauser comorbidities	457 (80.9)	2681 (78.8)	0.266
Heart failure	369 (65.3)	2044 (60.1)	0.019
Atrial fibrillation	160 (28.3)	784 (23.1)	0.006
Chronic pulmonary disease	227 (40.2)	1317 (38.7)	0.512
Chronic kidney disease	214 (37.9)	1187 (34.9)	0.171
Diabetes	284 (50.3)	1631 (48.0)	0.309
STEMI	59 (15.2)	506 (14.1)	0.584
Medicare payer	426 (75.4)	2343 (68.9)	0.002
*Hospital ownership*			
Government	71 (12.6)	332 (9.8)	< 0.001
Non-profit	451 (79.8)	2480 (72.9)	
Private	43 (7.6)	589 (17.3)	
*Hospital region*			
Northeast	111 (19.6)	544 (16.0)	< 0.001
Midwest	152 (26.9)	738 (21.7)	
South	193 (34.2)	1434 (42.2)	
West	109 (19.3)	685 (20.1)	
*Hospital bed size*			
Small	116 (20.5)	687 (20.2)	0.757
Medium	164 (29.0)	1040 (30.6)	
Large	285 (50.4)	1674 (49.2)	
Urban teaching hospital	433 (76.6)	2366 (69.6)	< 0.001

*IQR* interquartile range, *RSV* respiratory syncytial virus, *STEMI* ST-elevation myocardial infarction

Chi-square or Fisher’s Exact tests were used to compare categorical variables between cohorts. Continuous variables were compared via Mann–Whitney U tests. Adjusted analyses of dichotomous outcomes utilized logistic regression; while adjusted LOS comparisons were made via a generalized linear regression model with gamma-distributed error/log-link to account for the distribution of LOS. Statistical analyses were completed in SPSSv30 (IBM Corp., Armonk, NY). Our project was submitted to our institutional review board (IRB) as non-human subjects research and was acknowledged by our IRB as meeting criteria for this categorization.

## Results

We identified 3,966 MI admissions between 2016 and 2022 with either a coexisting RSV (*n* = 565) or influenza (*n* = 3401) infection in the dataset (Table 1). The median age for RSV and influenza patients was 74 (IQR = 65–82) and 72 (IQR = 63–81) years, respectively. The number of comorbidities did not differ significantly between cohorts. Of the MIs identified in this study, 15.2% (*n* = 59) within the RSV cohort and 14.1% (*n* = 506) within the influenza cohort were classified as STEMIs.

In-hospital mortality occurred in 5.7% of RSV patients and 5.8% of influenza patients (absolute difference = 0.1%; 95% confidence interval =  − 1.93% to 2.19%). Mean LOS for coexisting RSV and influenza patients was 7.2 ± 7.0 days and 6.9 ± 7.3 days, respectively. After adjusting for covariates, RSV was not associated with differences in in-hospital mortality (odds ratio = 0.925; 95% confidence interval = 0.620 to 1.382), in-hospital complications (odds ratio = 0.973; 95% confidence interval = 0.738 to 1.282), or LOS (mean difference = 0.17; 95% confidence interval =  − 0.30 to 0.64) compared to influenza (Table 2). No significant differences between cohorts were observed across sensitivity and subgroup analyses (Table 3).

**Table 2:** Outcomes associated with concomitant respiratory syncytial virus vs influenza infections

Outcome	RSV *N* = 565	Influenza *N* = 3401	Adjusted effect size^a^ (95%CI)
*Odds ratio*			
In-hospital mortality (%)	5.7%	5.8%	0.925 (0.620–1.382)
In-hospital complications (%)^b^	14.2%	13.9%	0.973 (0.738–1.282)
*Mean difference*			
LOS, days, mean ± SD	7.2 ± 7.0	6.9 ± 7.3	0.17 (− 0.300 to 0.640)

*CI* confidence interval, *LOS* length of stay, *RSV* respiratory syncytial virus, *SD* standard deviation

^a^All analyses adjusted for age, sex, Elixhauser comorbidities, atrial fibrillation, month of presentation, presentation before vs after 2020, STEMI, payer, hospital ownership, region, hospital teaching status

^b^In-hospital complications included cardiogenic shock, sepsis, mechanical ventilation, and cardiac arrest

**Table 3:** Sensitivity and subgroup analyses comparing respiratory syncytial virus vs influenza infections

Outcome	Adjusted effect size (95%CI)
***Analysis of patients admitted between 2016 and 2019, N* = *2643***	
Odds ratio-In-hospital mortality	**0.972 (0.562–1.681)**
Odds ratio-In-hospital complications	**0.857 (0.587–1.252)**
Mean difference-LOS	**0.160 (−0.790–0.460)**
***Analysis of patients admitted between 2020 and 2022, N* = *1323***	
Odds ratio-In-hospital mortality	**0.862 (0.470–1.579)**
Odds ratio-In-hospital complications	**1.125 (0.735–1.723)**
Mean difference-LOS	**0.600 (−0.030–1.220)**
***Analysis excluding patients with COVID-19 infections, N* = *3929***	
Odds ratio-In-hospital mortality	**0.982 (0.659–1.463)**
Odds ratio-In-hospital complications	**0.936 (0.708–1.238)**
Mean difference-LOS	**0.070 (−0.400–0.540)**

*CI* confidence interval, *LOS* length of stay

## Discussion

In this analysis of almost 4000 hospitalized MI patients, patient characteristics between those with a coexisting RSV infection were similar to those with a coexisting influenza infection. Observed in-hospital outcomes (e.g., mortality, LOS) were not meaningfully different between cohorts. The lack of observed differences in outcomes between the influenza and RSV cohorts should be considered in the context of well-established evidence that influenza adversely affects the prognosis of patients with MI [9].

Influenza has been associated with increased rates of cardiovascular events [1]. Studies have found upwards of a sixfold increase in the risk of an acute myocardial infarction (MI) within a week of a positive influenza test result [1]. Of the 1 billion people who contract influenza annually worldwide [10], 2.19% are at risk of experiencing a subsequent acute MI [11]. This incidence is also reportedly higher in those who have never been previously hospitalized for coronary artery disease compared to those who have [12]. Studies have also shown that mortality rates due to ischemic heart disease were increased during weeks of influenza epidemics [13]. Moreover, the influenza vaccine is known to be cardioprotective. A meta-analysis has demonstrated a relative risk reduction of 28%, 18%, and 13% for all-cause mortality, cardiovascular mortality, and adverse cardiovascular events, respectively, in vaccinated patients with preexisting cardiovascular disease [14]. In fact, studies have demonstrated potential benefits of influenza vaccination as part of in-hospital management of acute MI [14]. The American College of Cardiology (ACC)/American Heart Association (AHA) and European Society of Cardiology (ESC) have both endorsed annual influenza vaccination for patients with coronary artery disease and for all patients with acute coronary syndromes, preferably during the index hospitalization [15, 16].

A causal link between influenza and acute MI has been heavily hypothesized, although not confirmed. It is well understood that influenza infection can induce a septic state, in which symptoms such as fever, tachycardia, and hypoxemia are thought to cause an imbalance of myocardial oxygen supply and demand, leading to acute MI [2]. Another theory suggests that infection-induced inflammation can lead to plaque rupture, in addition to a prothrombotic state produced by platelet activation and endothelial dysfunction, all of which can result in thrombus formation and eventual acute MI [2].

Previous studies have investigated the relationship between RSV and the incidence of acute MI in older populations [17, 18]. In one such study, it was shown that 3.3% of older adults hospitalized for RSV in Italy had an acute MI [17]. A different study demonstrated that 10.9% of unvaccinated patients hospitalized for RSV had an acute cardiovascular event and that these patients were in fact at higher risk than those hospitalized for COVID-19 and influenza [18]. This suggests that like influenza infections, RSV infections may also be linked to hypoxia that disrupts myocardial oxygen demand and inflammation that promotes a prothrombotic state [2] and may explain why no difference in outcomes was observed in our study. The aforementioned studies and theoretical impact of RSV have led societies like the ACC/AHA and ESC to begin to discuss the conceptual benefits of RSV vaccination among patients with cardiovascular disease [15, 16]. Our current work also supports this. Similarly, given the aforementioned evidence and our own findings, RSV may need to be considered in future evaluations of risk-stratification when patients present with MI.

The RSV vaccine was first made available in 2023, and the novelty of the RSV vaccine affects the applicability of study results [2]. As a result, lack of RSV vaccination in the study population may have inflated the degree of the association between RSV and acute MI outcomes. This is notable as it is unlikely that trends in vaccine availability after 2023 influence the influenza cohort to the same extent as the RSV cohort. Moreover, previous studies have shown that vaccination for respiratory viruses decreases cardiovascular morbidity [2, 14, 18]. Increased RSV vaccination of older adults in the future has the potential to mitigate the outcomes measured in our current study [2]; however, the true impacts will not be known until a meaningful amount of newer data that include RSV vaccination status is available.

In addition to the aforementioned limitation related to RSV vaccination status, our study had some other limitations. We were unable to evaluate post-discharge outcomes, such as re-admission, development of additional major adverse cardiac events (MACE) or the development or exacerbation of heart failure. These post-hospitalization outcomes are clinically meaningful, as they may influence long-term prognosis, functional status, and health-related quality of life [1, 2, 9, 17, 18]. As a result, our findings are limited to in-hospital outcomes and may underestimate the full clinical impact of viral co-infections in patients with MI. Future studies incorporating extended follow-up and outpatient data are needed to better characterize the long-term consequences of viral co-infections. Similarly, we were not able to evaluate common hospital complications that can also be coded as comorbidities, such as acute arrhythmias or acute heart failure exacerbations, due to the cross-sectional nature of the data and the absence of data beyond administrative sources. Due to the cross-sectional nature of the data, we were not able to evaluate the temporal relationship between symptoms of MI and the viral infections. We were also not able to evaluate how closely patients were followed by healthcare teams prior to the MI. Patients with better preventative care prior to their MI would be expected to have better health outcomes and we were not able to measure if preventative care differed between cohorts.

Our study identified viral infections based on diagnostic coding and not laboratory-confirmed infections, as the latter data point is not available in the NIS. It is possible that RSV infections are undercoded in administrative data. The reliance on diagnostic coding to identify infections may also have resulted in imperfect specificity, as some patients included in the dataset could have had suspected rather than confirmed infections. Similarly, guidance for documenting RSV infections was updated in 2020 and our study spanned from 2016 to 2022 [19]. It is possible that those identified in the RSV cohort prior to 2020 had more severe disease due to differences in coding practices. To address this and potential disruptions in care during the COVID-19 pandemic (e.g., care disruptions related to hospital capacity strain, including staffing shortages and limited bed availability), we included a year variable in our adjusted models and report results before and after 2020. No differences between cohorts were observed in the sensitivity/subgroup analyses. Interpretation of these results should account for the sample size and the frequency of mortality (~ 6%). Although the difference between groups was small (i.e., 0.1%), the confidence interval around this difference spanned from approximately −2% to 2%. Finally, we did not add treatment variables (i.e., percutaneous coronary intervention, coronary artery bypass grafting, thrombolysis) in our model, as these occur after the presentation and adjusting for them may risk removing part of the presentation’s effect or creating new bias.

In conclusion, our study found no significant difference in in-hospital mortality or LOS between patients with MI coexisting with RSV and those with influenza infections. Given the established role of influenza in exacerbating MI severity, greater recognition and investigation of RSV co-infections in this high-risk population are likely needed.

## Data Availability

Data used in this study are available via the Healthcare Cost and Utilization Project.

## References

[1] Kwong JC, Schwartz KL, Campitelli MA et al (2018) Acute myocardial infarction after laboratory-confirmed influenza infection. N Engl J Med 378(4):345–353 29365305 10.1056/NEJMoa1702090

[2] Tanriover MD, Azap A, Cakir Edis E et al (2025) Respiratory syncytial virus (RSV) infections in adults: Current trends and recommendations for prevention - a global challenge from a local perspective. Hum Vaccin Immunother 21(1):2514357 40530658 10.1080/21645515.2025.2514357 PMC12184840

[3] Agency for Healthcare Research and Quality (AHRQ). HCUP National Inpatient Sample (NIS). Healthcare Cost and Utilization Project (HCUP). Rockville, MD. Published annually. Accessed January 31, 2026. https://hcup-us.ahrq.gov/nisoverview.jsp

[4] Patel AB, Quan H, Welsh RC et al (2015) Validity and utility of ICD-10 administrative health data for identifying ST- and non-ST-elevation myocardial infarction based on physician chart review. CMAJ Open 3(4):E413–E418 27570759 10.9778/cmajo.20150060 PMC4987155

[5] Movva N, Suh M, Reichert H et al (2022) Respiratory syncytial virus during the COVID‑19 pandemic compared to historic levels: a retrospective cohort study of a health system. J Infect Dis 226(Suppl 2):S175–S183 35968868 10.1093/infdis/jiac220 PMC9377040

[6] Xie Y, Choi T, Al‑Aly Z (2024) Long-term outcomes following hospital admission for COVID‑19 versus seasonal influenza: a cohort study. Lancet Infect Dis 24(3):239–255 38104583 10.1016/S1473-3099(23)00684-9

[7] Cai W, Tolksdorf K, Hirve S et al (2020) Evaluation of using ICD-10 code data for respiratory syncytial virus surveillance. Influenza Other Respir Viruses 14(6):630–637 31206246 10.1111/irv.12665 PMC7578302

[8] Fingar KR, Liang L, Stocks C. Diagnosis codes defining influenza – Inpatient Hospital Stays and Emergency Department Visits Involving Influenza, 2006–2016. Healthcare Cost and Utilization Project (HCUP) Statistical Briefs [Internet]. Agency for Healthcare Research and Quality (US). Accessed July 1, 2025 https://www.ncbi.nlm.nih.gov/books/NBK550181/table/sb253.tab8/

[9] Tripathi B, Kumar V, Kalra A et al (2020) Influence of influenza infection on in-hospital acute myocardial infarction outcomes. Am J Cardiol 130:7–14 32636019 10.1016/j.amjcard.2020.05.045

[10] World Health Organization. (2025, February 28). Influenza (seasonal). Accessed June 1, 2025 https://www.who.int/news-room/fact-sheets/detail/influenza-(seasonal)#:~:text=There%20are%20around%20a%20billion,infections%20are%20in%20developing%20countries

[11] Ouranos K, Vassilopoulos S, Vassilopoulos A, Shehadeh F, Mylonakis E (2023) Cumulative incidence and mortality rate of cardiovascular complications due to laboratory‐confirmed influenza virus infection: a systematic review and meta‐analysis. Rev Med Virol 34(1):2497 10.1002/rmv.2497 38126946

[12] de Boer AR, Riezebos-Brilman A, van Hout D, van Mourik MSM, Rümke LW, de Hoog MLA, Vaartjes I, Bruijning-Verhagen PCJL (2024) Influenza infection and acute myocardial infarction. NEJM Evidence 3(7):EVIDoa2300361 38916418 10.1056/EVIDoa2300361

[13] Warren-Gash C, Smeeth L, Hayward AC (2009) Influenza as a trigger for acute myocardial infarction or death from cardiovascular disease: a systematic review. Lancet Infect Dis 9(10):601–610 19778762 10.1016/S1473-3099(09)70233-6

[14] Bocale R, Necozione S, Desideri G (2022) The link between influenza and myocardial infarction: vaccination protects. Eur Heart J Suppl 24(Suppl I):I84–I88. 10.1093/eurheartjsupp/suac078 36380797 10.1093/eurheartjsupp/suac078 PMC9653152

[15] Heidenreich PA, Bhatt DL, Nazir NT et al (2025) 2025 ACC expert consensus statement on adult immunizations as part of cardiovascular care: a report of the American College of Cardiology Solution Set Oversight Committee. J Am Coll Cardiol 76(9):1097–1125 10.1016/j.jacc.2025.07.003 40856667

[16] Heidecker B, Libby P, Vassiliou VS et al (2025) Vaccination as a new form of cardiovascular prevention: a European Society of Cardiology clinical consensus statement. Eur Heart J 46(36):3518–3531. 10.1093/eurheartj/ehaf384 40582710 10.1093/eurheartj/ehaf384

[17] Puggina A, Dovizio M, Domnich A et al (2025) Demographics and clinical burden of disease among RSV-hospitalized older adults in Italy: a retrospective cohort study. Hum Vaccin Immunother 21(1):2479334 40126050 10.1080/21645515.2025.2479334 PMC11934162

[18] Wee LE, Lim JT, Ho RWL, Chiew CJ, Lye DCB, Tan KB (2025) Cardiac events in adults hospitalized for respiratory syncytial virus vs COVID-19 or influenza. JAMA Network Open. 8(5):e2511764 40402498 10.1001/jamanetworkopen.2025.11764 PMC12100453

[19] Centers for Medicare & Medicaid Services (CMS), National Center for Health Statistics (NCHS). ICD‑10‑CM Official Guidelines for Coding and Reporting, FY 2020 (October 1, 2019–September 30, 2020). U.S. Department of Health and Human Services; 2019. Accessed February 1, 2026. https://www.cdc.gov/nchs/data/icd/10cmguidelines-FY2020_final.pdf

